# Evaluation of Touch and Durability of Cotton Knit Fabrics Treated with Reactive Urethane-Silicone Softener

**DOI:** 10.3390/polym14091873

**Published:** 2022-05-03

**Authors:** Hang Sung Cho, Hye Jun Yoon, Bum Hoon Lee, Jang Chang Woo, Hyeong Yeol Choi, Euijin Shim, Ji Ho Youk

**Affiliations:** 1 Advanced Textile R&D Department, Korea Institute of Industrial Technology (KITECH), Ansan-si 15588, Korea; hscho@kitech.re.kr (H.S.C.); previa@kitech.re.kr (H.J.Y.); woocatcher@kitech.re.kr (J.C.W.); 2 Department of Textile Materials Engineering, Shinhan University, Dongducheon-si 11340, Korea; bhlee@shinhan.ac.kr; 3 Department of Fashion Design, Dong-A University, Busan 49315, Korea; spaachoi@dau.ac.kr; 4 Department of Chemistry and Chemical Engineering, Education and Research Center for Smart Energy and Materials, Inha University, Incheon 22212, Korea

**Keywords:** amino silicone softener, blocked isocyanate, KES-FB system, washing durability, dimensional stability

## Abstract

A new reactive urethane–silicone softener was developed to provide a soft touch to cotton knit fabrics with improved durability to washing and dimensional stability. The reactive urethane–silicone softener consisted of an amino silicone softener and a blocked isocyanate, which can crosslink and react with cellulose surfaces. The activated isocyanate from the blocked isocyanate reacted with the amino silicone softener by heat treatment at 150 °C for 30 min. The mechanical properties of the cotton knit fabrics treated with the urethane–silicone softener were evaluated using a Kawabata Evaluation System-Fabrics (KES-FB) system. The cotton knit fabrics treated with the urethane–silicone softener showed excellent elasticity, flexibility and shear recovery as well as excellent recovery against bending deformation, and soft and smooth surface characteristics with a small coefficient of friction that were maintained even after washing 20 times.

## 1. Introduction

As living standards improve and textile product consumption becomes more advanced, diversified, and differentiated, knitted cellulose products are considered high-quality premium products, and consumer demand is increasing [1,2,3,4]. Knitted products made from cellulose-based materials, such as cotton, rayon, and modal, are luxurious and flexible, giving a comfortable feel. On the other hand, knitted fabrics lack dimensional stability compared to woven fabrics [5,6,7]. Accordingly, continuous research is being conducted on developing and processing elastic softeners for knitted products using cellulose to improve the dimensional stability and commercialization of these cellulose knit products as high-quality premium products [8,9,10,11,12,13,14].

Fabric softeners are used as additives and for in-home laundering in wet textile processing to improve fabric handling. Conventional softeners do not chemically react with cotton fibers, so there is a limit to improving the washing durability and dimensional stability of cotton knitwear. Nevertheless, these softeners adhere to the fiber surface usually through a weak electrical attraction without any chemical bonds, causing poor washing durability. On the other hand, durable silicone-based fabric softeners render additional performance properties to cotton fabric, such as improved wrinkle recovery and crease resistance, and improved wear comfort with a smooth handle [15].

Several previous studies have used hydrated amino silicone oil to improve the reactivity of cellulosic fibers with softeners. In these studies, the researchers emulsified the oil into small, stable nanoparticles by crosslinking, and reported improved washing durability, dimensional stability, and yellowing [16,17]. Nevertheless, some emulsified particles may remain on the fabric surface and can be partially concentrated, resulting in a white coating in the form of aggregation rings [18,19].

In this study, to improve these drawbacks, a reactive urethane–silicone softener was developed to impart a flexible feel to cellulose knitwear with enhanced washing durability and dimensional stability. A urethane–silicone softener was prepared by mixing a blocked isocyanate with an amino silicone softener to react with the fiber surface and the softener. Because the reaction of the blocked isocyanate occurs only in the finishing process, the disadvantage due to the rubber effect is improved when using a two-component treatment agent in which a conventional silicone softener and water-soluble urethane are mixed. In addition, to quantify the sensory characteristics of the flexible finish of the softening agent-treated cellulose knitted fabric and evaluate the sensitivity, KES-FB (Kawabata Evaluation System-Fabrics) was used to measure the objective mechanical properties of the cotton knitted fabric processed with the urethane–silicone softener [20,21,22]. The urethane–silicone softener-treated cotton fabrics exhibited excellent elasticity, flexibility, and shear recovery after 20 washes, as well as excellent recovery against bending deformation, and soft and smooth surface properties with a small coefficient of friction.

## 2. Materials and Methods

### 2.1. Materials

2,4,6-Trioxotriazine-1,3,5(2H,4H,6H)-triyl)tris(hexamethylene) isocyanate (HDI-trimer, Vencorex, Incheon, Korea, 99.0%), 1,6-hexandiol (HD, Daejung chemical, Siheung-si, Korea, 99.5%), and poly(ethylene) glycol methyl ether (MPEG, IC chemical Korea, Yeosu, Korea, 98.0%) were used to synthesize the urethane prepolymer. A low-temperature curing type 3,5-dimethylpyrazole (DMP, Daejung chemical, 99%) was used to synthesize the blocked isocyanate. The knit used in the flexible processing experiment is a 30-count single cotton knitted fabric. After refining and bleaching, Turquoise blue dye (Suncion T/Blue HA, Ohyoung, Seoul, Korea) was used at a concentration of 1.0% o.w.f at 80 °C, and a dyeing time of 60 min. It was dyed with a pilot.

### 2.2. Development of Urethane–Silicone Softener

#### 2.2.1. Synthesis of Blocked Isocyanate

HDI-trimer, HD, and MPEG were added to a four-neck round bottom flask with a reflux condenser attached at a molar ratio of 2/1/1 to synthesize the urethane prepolymer through a reaction. DMP was added at a molar ratio of three to act as a blocking agent to the prepared urethane prepolymer, causing a reaction that theoretically synthesized a blocked isocyanate with the isocyanate (–NCO) group completely blocked off.

#### 2.2.2. Urethane–Silicone Softener Manufacturing

Blocked isocyanate emulsions (0.5 wt.%) were first prepared by stirring the blocked isocyanate in water at 2000 rpm for 30 min using a homogenizer. The blocked isocyanate emulsion was blended into a commercial amino silicone softener (E-silky, Hyundae Hichem) to manufacture the urethane–silicone softener. The commercial amino silicone softener was prepared by the emulsion polymerization of hydrophilized amino-based silicone oil with a 0.9% amine content, consisting of dispersive particles with a particle size of 100 to 150 nm.

#### 2.2.3. Determination of NCO Content

A 2 g sample was added to a 300 mL triangular flask, and 25 mL of an *n*-butylamine/toluene solution at a molar concentration of one was added. The mixture was then shaken gently for 15 min. Subsequently, 150 mL of isopropyl alcohol and 0.5 mL of the indicator bromocresol were added and mixed. Titration was performed with a 0.5 N hydrochloric acid solution. The endpoint was the point where the blue or blue-violet color of the solution disappeared, and the yellow color lasted for more than 15 s. Finally, the –NCO content was determined using the following equation [23,24,25]:

$$\mathrm{NCO}\left(\%\right)=\frac{\left(B-S\right)\times 42\times N\times f}{w\times 1000}\times 100\left(\%\right)$$

*B*: amount of 0.5 N hydrochloric acid solution by blank test (mL).
*S*: amount of 0.5 N hydrochloric acid solution used in the sample (mL).
42: molecular weight of NCO (g/mol).
*N*: normal concentration (N) of the hydrochloric acid solution used.
*f*: factor of 0.5 N hydrochloric acid solution.
*w*: mass of sample (g).

### 2.3. Softening Finishes

Pre-treated and dyed 30 s single cotton knit (32G/G, 130 g) was treated with a softener diluted to 30 g/L, padded with a horizontal padder (DL-2005H, Daelim starlet, Siheung-si, Korea) with a pickup ratio of 120% and padding pressure of one bar, and dried at 120 °C for 2 min to cure the fabric at 160 °C for another 2 min.

### 2.4. Characterization

#### 2.4.1. Particle Size Analysis

A laser scattering particle size distribution analyzer (LA950, Horiba, Kyoto, Japan) was used to measure the average particle size of a softener emulsifier.

#### 2.4.2. Fourier Transform Infrared (FTIR) Analysis

FTIR (Nicolet i5, Thermo Scientific, Waltham, MA, USA) spectroscopy was conducted to confirm the progress of the reaction according to reaction temperature and time.

### 2.5. Evaluation of Mechanical Properties and KES-FB System Analysis of Cotton Knitwear Treated with Urethane–Silicone Softener

The mechanical properties of cotton knitwear were compared before and after treatment with an amino silicone softener (Sil-softer SB, Hyundae Hichem, Siheung-si, Korea) as a reference and a urethane–silicone softener. The mechanical properties of the cotton knit fabric processed with a softener were measured using the KES-FB system for tensile, bending, surface, shear, and compression properties, weight, and thickness under the given conditions listed in Table 1. All samples were cut to a 20 cm × 20 cm size. The measured data were used by averaging the warp and weft wale results. In addition, the changes in the mechanical properties of cotton knitwear after washes were also compared to confirm the washing durability of the softener. The washing conditions were as follows: 66 g of detergent (WOB, AATCC, Durham, NC, USA) was added to the softening agent-treated cotton knitted fabric under normal general conditions, washed 20 times in a Canmore washing machine, and dynamic properties were measured under the same conditions.

## 3. Results and Discussion

### 3.1. Synthesis of Blocked Isocyanate

**Figure 1:**
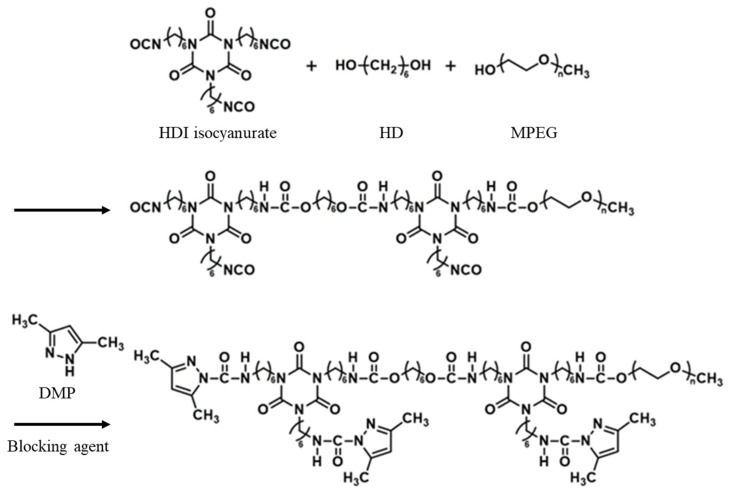
Synthesis scheme of urethane prepolymer and blocking of the residual isocyanate groups using a blocking agent.

A blocked isocyanate was synthesized to develop a reactive urethane–silicone softener and improve the feel and durability of cotton fabric. For the synthesis of blocked isocyanate, HDI-trimer, HD, and MPEG were reacted at a molar ratio of 2/1/1 to polymerize urethane prepolymer, as shown in Figure 1. The number of moles of the –NCO group was more significant than the number of moles of the added hydroxyl group (-OH) of HD and MPEG. Hence, the –NCO group that did not undergo a reaction remained in the urethane prepolymer. Subsequently, the DMP blocker reacted with the remaining –NCO group, resulting in a blocked isocyanate, where the –NCO group was sealed [26,27,28].

**Figure 2:**
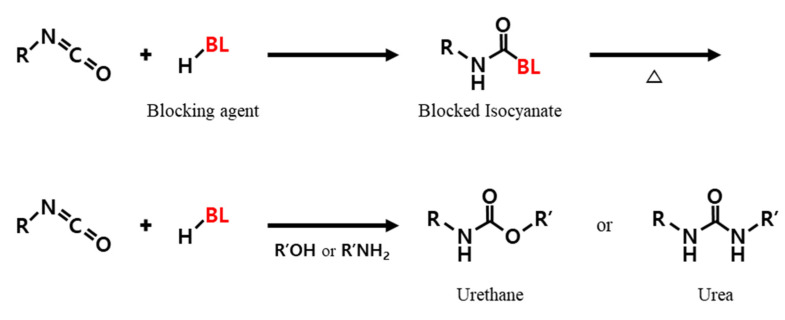
Blocking and reactivation reaction mechanism of the isocyanate groups.

**Figure 3:**
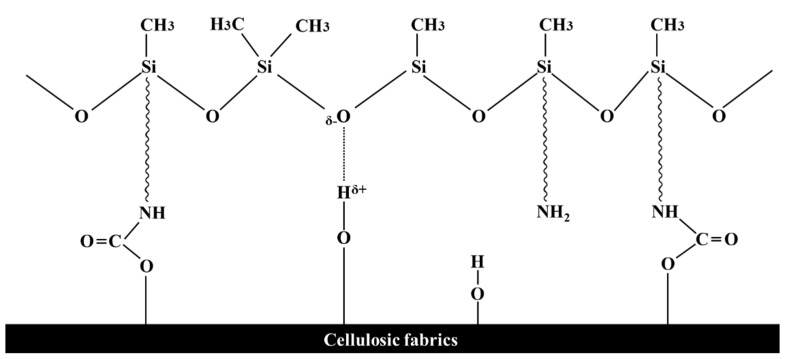
Crosslinking chemical mechanism of cellulosic fabrics and softeners.

**Figure 4:**
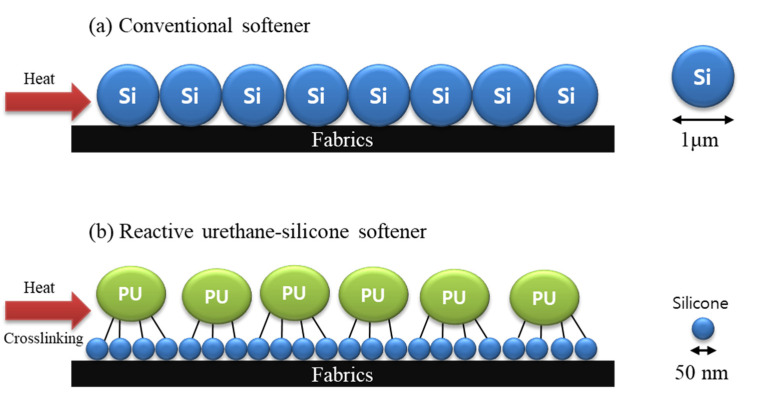
Schematic of conventional softener and reactive urethane-silicone softeners. (**a**) conventional softener; (**b**) reactive urethane-silicone softener.

Figure 2 presents a schematic diagram of the sealing reaction that deactivates the residual –NCO group and reactivates the –NCO group during heat treatment. The residual –NCO group was sealed using blockers for stability and storage while processing the obtained urethane prepolymer. The reactivated –NCO group can react with the –OH group to form urethane bonds or a –NH_2_ group to form urea bonds [26,29]. Figure 3 presents a schematic diagram of the reaction between the cellulose fabric and the developed softener. Figure 4 schematically shows the particle size and reaction of the conventional softener and the reactive urethane–silicone softener on the fabric surface. Particles that are 1/20 times smaller than conventional particles can increase surface area and surface reactivity. Further, since the particle size of the softener is small, there is an advantage in that the permeability of the fabric is improved and the durability is improved [8]. In addition, it has morphological stability due to the crosslinking reaction with urethane.

**Figure 5:**
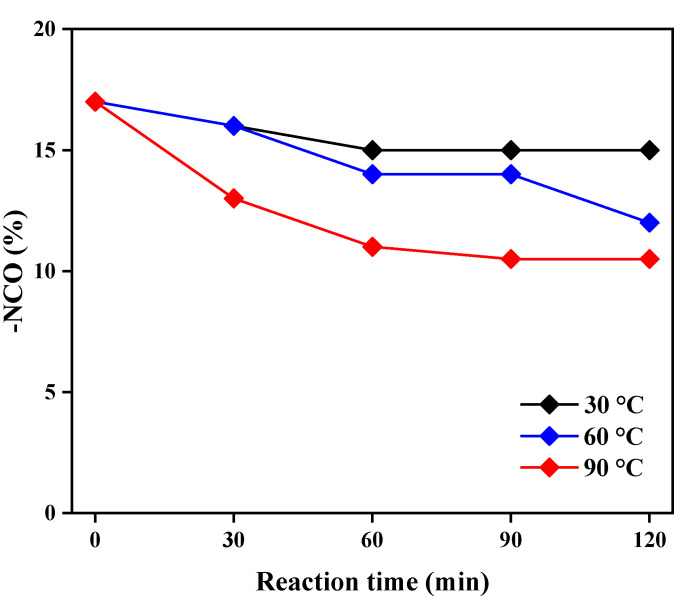
Changes in the –NCO group content with the reaction time and temperature.

Figure 5 shows the change in the –NCO group content according to the reaction time determined by titration of the urethane prepolymer. The change in the content of–NCO groups according to the reaction time was similar at 30 and 60 °C, but the content of –NCO groups according to reaction time at 90 °C decreased significantly. Therefore, the reaction temperature of the HDI-trimer, HD, and MPEG for the synthesis of the urethane prepolymer was determined to be 90 °C, with a reaction time of 90 min [30].

**Figure 6:**
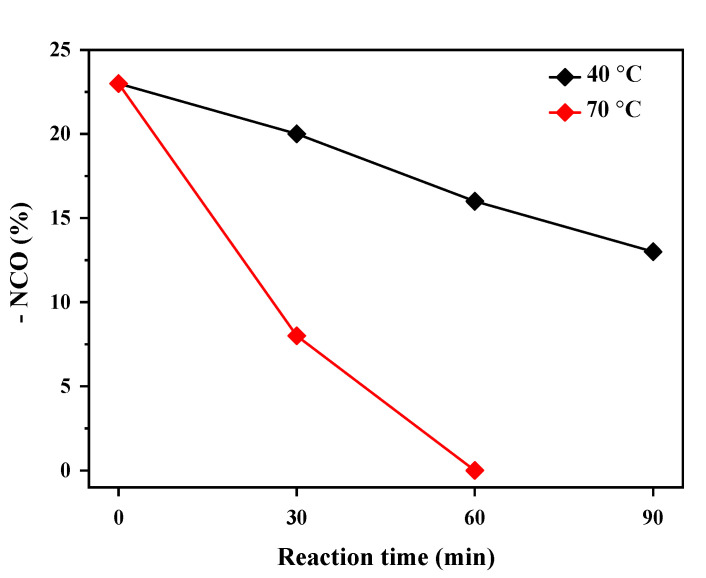
Changes in –NCO group content with the reaction of the urethane prepolymer and DMP.

Figure 6 shows changes in the –NCO content according to the reaction time and temperature of a urethane prepolymer and DMP. This study used DMP, a low-temperature curing type, with an activation temperature of 110 to 120 °C as a blocking agent. The urethane prepolymer and DMP contained –NCO groups that did not react with DMP after 90 min reaction time at 40 °C, but very little –NCO remained after 60 min reaction time at 70 °C. Therefore, the basic reaction conditions for urethane prepolymer and DMP were determined to be a reaction temperature of 70 °C with a reaction time of 60 min.

### 3.2. Characterization of Urethane–Silicone Softener

A blocked isocyanate emulsion (0.5 wt.%) was prepared by first stirring the blocked isocyanate in water at 2000 rpm for 30 min using a homogenizer. The urethane–silicone softener was prepared by blending 0.5% by weight of a blocked isocyanate emulsion with a commercially available amino silicone softener (E-silky, Hyundai Hichem, Siheung-si, Korea). The particle size of the prepared blocked isocyanate emulsion was 0.059 nm on average, as shown in Table 2.

**Figure 7:**
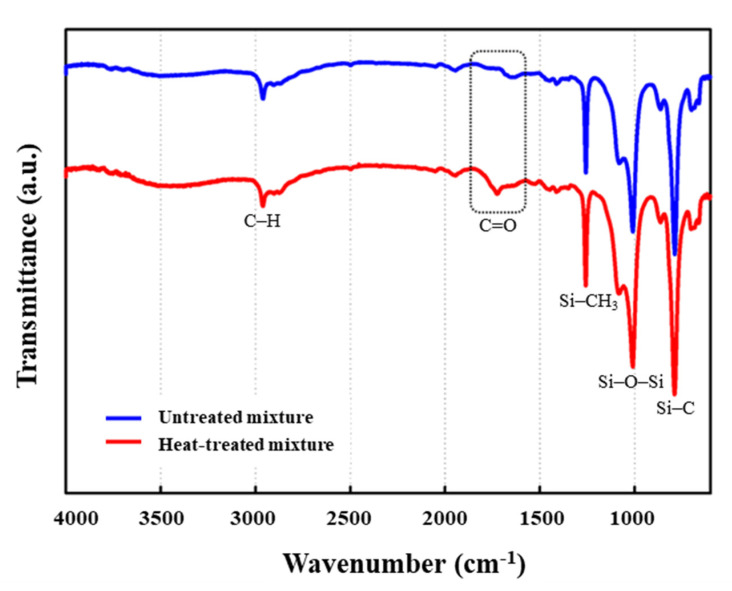
FTIR spectra of a mixture of amino silicone softener and blocked isocyanate before (blue color) and after (red color) drying at 150 °C for 30 min.

Figure 7 presents the FTIR spectra of a mixture of the amino silicone softener and the blocked isocyanate before and after drying at 150 °C for 30 min. After heat treatment, multiple absorption bands were observed in the C=O regions (1700 cm^−1^). The changes were related to hydrogen bonding of the urethane or urea groups [31]. This is the peak that shows that the crosslinking reaction between blocked isocyanate and amino silicone has occurred. As suggested in Figure 2, the heat treatment induced a reaction between the emulsified blocked isocyanate and the amino silicone softener. The hydroxyl groups (–OH groups) on the surface of the cotton fabric can react with blocked isocyanates to improve the wash durability and dimensional stability of cotton knitwear. The –NCO group of the isocyanate is regenerated after unblocking, and a urethane bond is formed with the –OH group of the cellulose fabric. This chemical bond provides a strong covalent bond between the fabric and the softener. In this way, the isocyanate acts as a coupling agent for fabric surface modification [32,33].

The peaks observed in both spectra regardless of heat treatment are as follows: The peak at 2960 cm^−1^ is attributed to the C–H stretch absorption of CH_3_ groups, and the 810 cm^−1^ band is due to Si–C [34,35]. Furthermore, the peak due to Si–O–Si asymmetric stretching, which generally appears in the range of 950 cm^−1^ to 1200 cm^−1^, was observed at 1075 cm^−1^, and Si–CH_3_ bonding due to CH symmetric strain was observed at 1250 cm^−1^ [36].

### 3.3. Evaluation of Mechanical Properties and KES-FB System Analysis

#### 3.3.1. Tensile Properties of Cotton Knitwear after Softener Treatment

**Figure 8:**
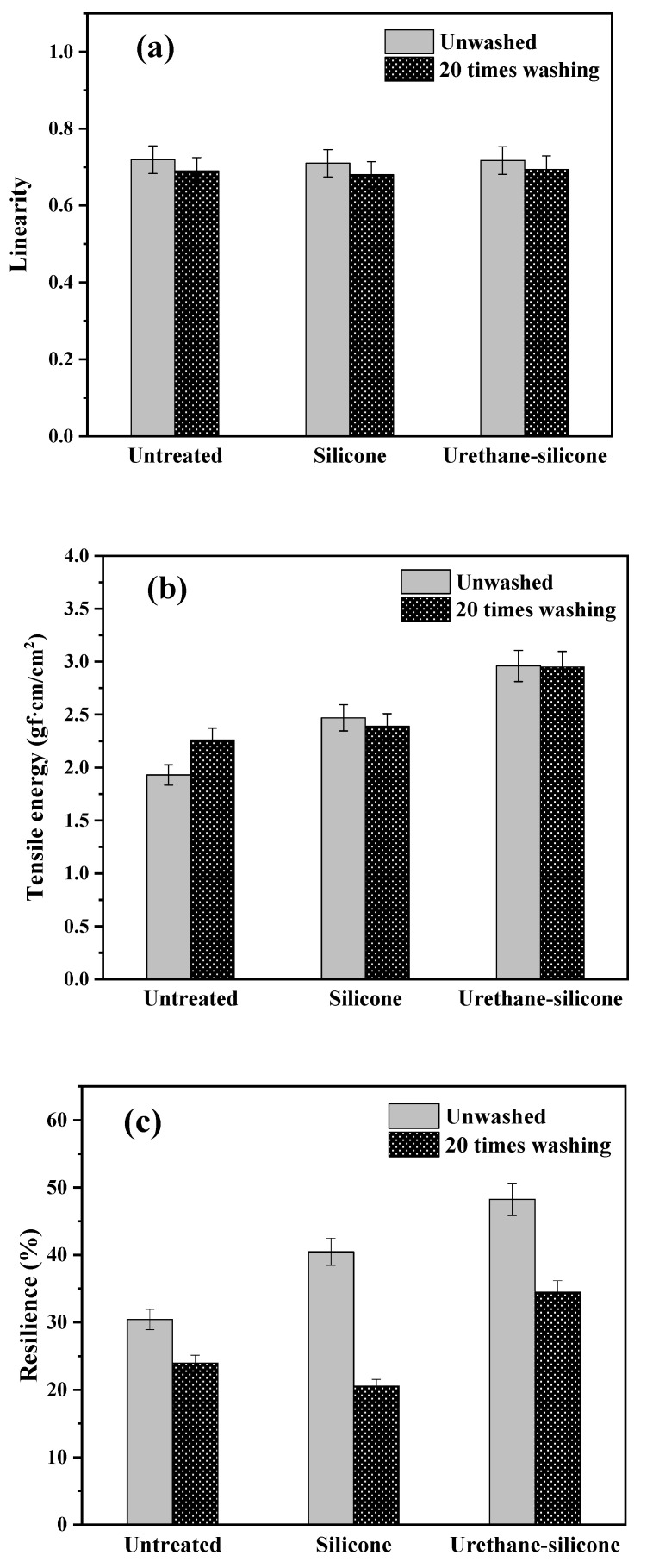
Tensile properties of the cotton knit fabric untreated and treated with softeners. (**a**) Linearity; (**b**) tensile energy; (**c**) resilience.

Figure 8 presents the result of the tensile properties of cotton knitwear treated with softeners. The linearity (LT), tensile energy per unit area (WT), and resilience (RT) were measured as tensile properties. The smaller the LT value, the easier the tensile deformation of fabric. Moreover, the greater the elasticity of the fabric, the greater the LT value [37]. The average LT values for the wale and course direction of the untreated cotton knitwear were similar to the values of cotton knitwear treated with the Sil-softer SB and urethane–silicone softener (Figure 8a). This is probably due to the excellent tensile properties that knits have inherently [38]. The LT values of the three-cotton knitwear showed similar values even after 20 washes, indicating excellent durability (Figure 8a). In other words, the chemical reaction between urethane–silicone and the fiber surface helped improve the overall tensile properties.

WT is the tensile energy during tensile deformation that occurs when weight is applied to the fabric, and the value of WT increases with increasing length. The WT value was the largest in cotton knitwear treated with the urethane–silicone softener (Figure 8b), indicating that when tensile energy is applied per unit area of WT, it is possible to show that the elasticity is good because it has the greatest value in the cotton knit treated with urethane–silicone softener.

RT represents recovery from tensile strain. The higher the RT value, the better the recovery [37]. Figure 8c shows that the cotton knitted fabric treated with the urethane–silicone softener recovers best after applying tensile strength. The average RT value of the three cotton knitted fabrics decreased significantly after washing 20 times. After washing, some of the softener has no choice but to fall off due to physical force [39,40,41,42]. Comparing the resilience after 20 washes, the resilience value was lower than that of untreated when using silicone softener, but it was the highest at 35% when using urethane–silicone softener. This value is more flexible than the untreated, unwashed sample.

#### 3.3.2. Shear Properties of Cotton Knitwear after Softener Treatment

**Figure 9:**
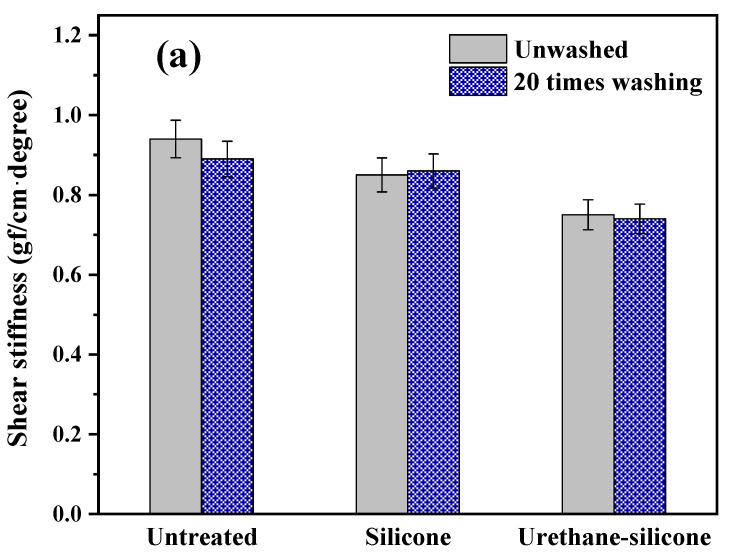

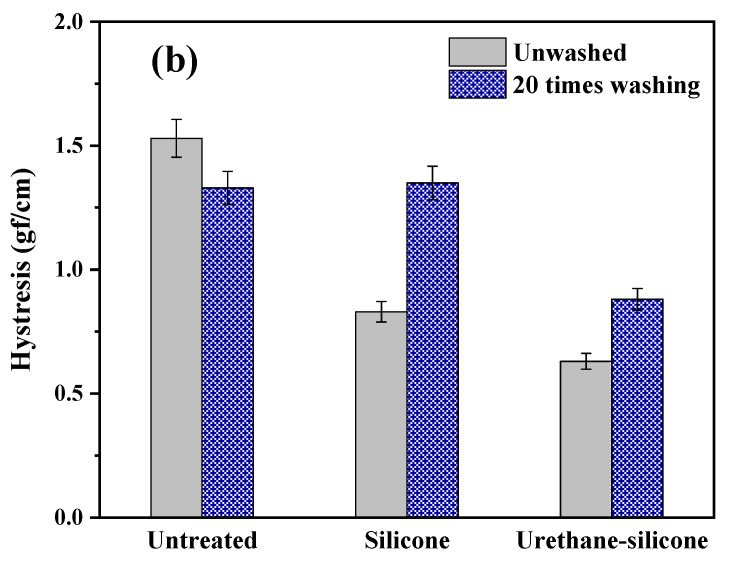
Shear properties of the cotton knit fabric untreated and treated with softeners. (**a**) Shear stiffness; (**b**) hysteresis.

Figure 9 presents the shear properties of cotton knitwear treated with a softener. The G value, which represents the shear rigidity, is affected by the contact pressure and friction between the yarns that make up the fabric. A lower G value means the fabric is deformed more easily in the diagonal direction. If deformation occurs easily in the diagonal direction, the person wearing the fabric can feel more comfortable with the movement. Cotton knitwear treated with the urethane–silicone softener had the smallest G value (Figure 9a), meaning that it was most prone to shear deformation. This tendency did not change even after washing 20 times.

A smaller 2HG value representing the history of shear rigidity indicated better resilience (recoverability) to shear deformation [37]. A comparison of the 2HG values in Figure 9b showed that the cotton knitwear treated with urethane–silicone softener showed the best recovery performance for shear deformation because of the smallest 2HG value. Overall, cotton knitwear treated with urethane–silicone softener showed excellent flexibility and resilience against shear stress. Therefore, applying softener appeared to have brought about a reduction in shear rigidity (G) and shear hysteresis (2HG) values, leading to the improvement of fabric handle and formability properties [43].

The increased 2HG value indicates that the washed fabric has poorer recovery than the untreated fabric [44]. The increase in the 2HG value after washing is believed to hinder recovery when shear deformation is applied due to the shrinkage of the sample by washing [45,46]. Thus, the chemical reaction between urethane–silicone and the fiber surface helps to improve the overall shear properties, such as the tensile properties.

#### 3.3.3. Bending Properties of Cotton Knitwear after Softener Treatment

**Figure 10:**
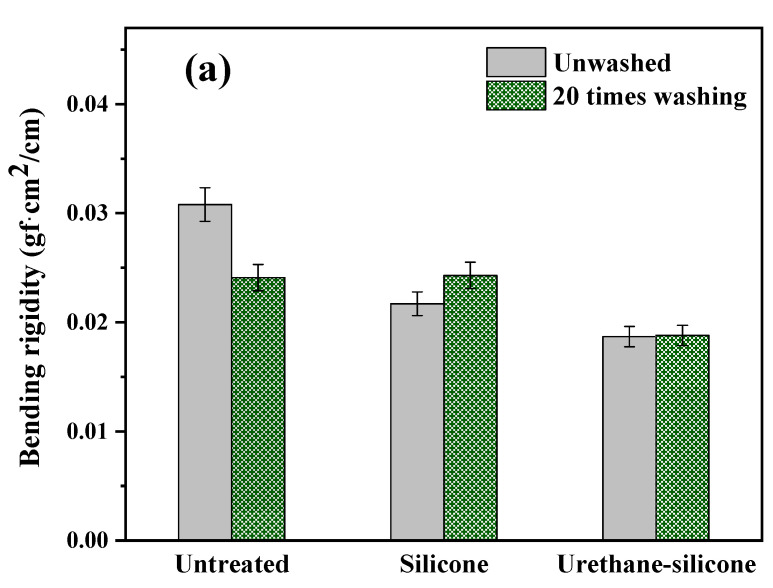

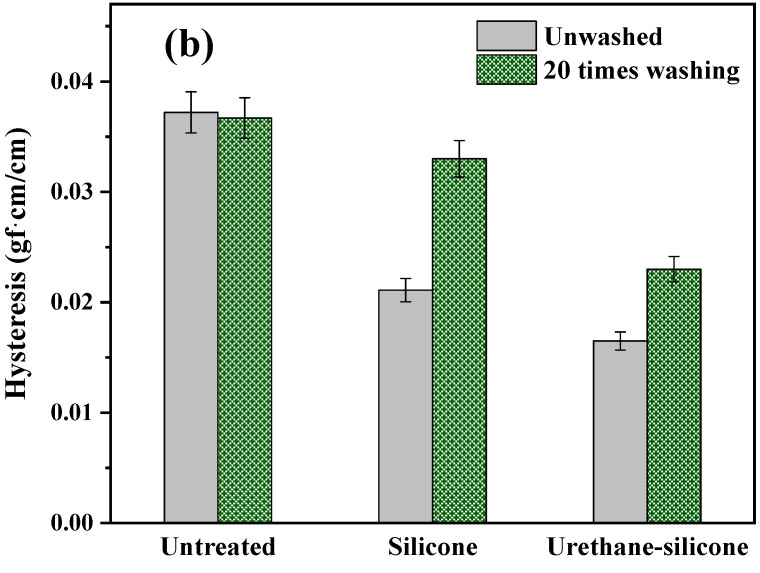
Bending properties of the cotton knit fabric untreated and treated with softeners. (**a**) Bending rigidity; (**b**) hysteresis.

Figure 10 shows the bending properties of cotton knitwear treated with a softener. The B value represents the bending rigidity, which demonstrates the stiffness of the fabric. The larger the value, the stiffer the fabric, and the smaller the value, the more flexible the fabric. A comparison of the B values revealed cotton knitwear treated with the urethane–silicone softener to have the smallest value (Figure 10a), demonstrating its excellent flexibility. This tendency did not change, even after washing 20 times. In addition, a smaller 2HB value indicates a history of bending deformation, which demonstrates excellent bending resilience. As a result of comparing the 2HB value, the cotton knitted fabric with the added urethane–silicone softener showed the smallest value for bending deformation, which was maintained even after washing 20 times, demonstrating that it had the greatest restoring force (Figure 10b). In conclusion, the urethane–silicone softener acts as a reinforcing material for the knitted fabric because of its flexibility and the elasticity of urethane and silicone due to the chemical bonding of the fabric surface [15,43,47,48].

#### 3.3.4. Compression Properties of Cotton Knitwear after Softener Treatment

**Figure 11:**
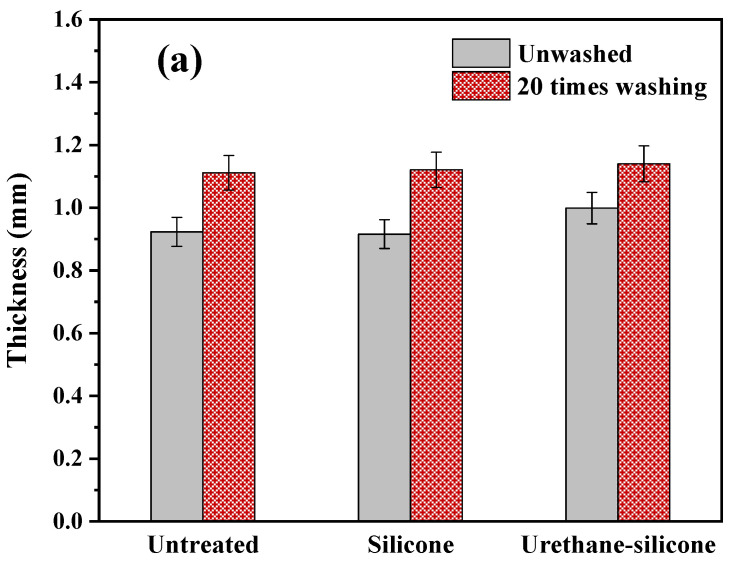

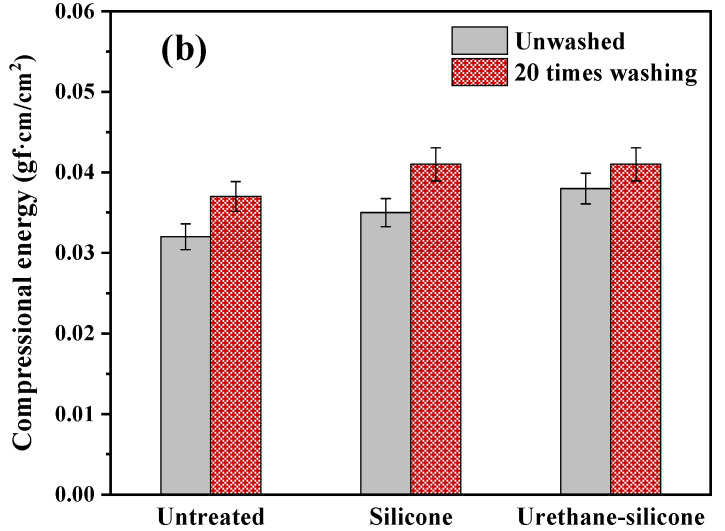
Compression properties of the cotton knit fabric untreated and treated with softeners. (**a**) Thickness; (**b**) compressional energy.

The compression properties are factors that can evaluate the volume properties of the fabric. Figure 11 shows the results of the compression properties of cotton knitwear treated with a softener. The T_0_ represents the thickness of the fabric, and WC represents the compression energy of the fabric.

A comparison of the fabric thickness revealed the urethane–silicone softener-treated cotton knitted fabric to be thicker (Figure 11a). The larger the WC value, the bulkier the fabric is. The WC value of cotton knitwear treated with the urethane–silicone softener was the largest (Figure 11b). The increase in physical thickness through silicone–urethane affects the compression properties. This suggests that the chemical reaction of the softener on the fiber surface may help improve the compression properties [47].

#### 3.3.5. Surface Properties of Cotton Knitwear after Softener Treatment

**Figure 12:**
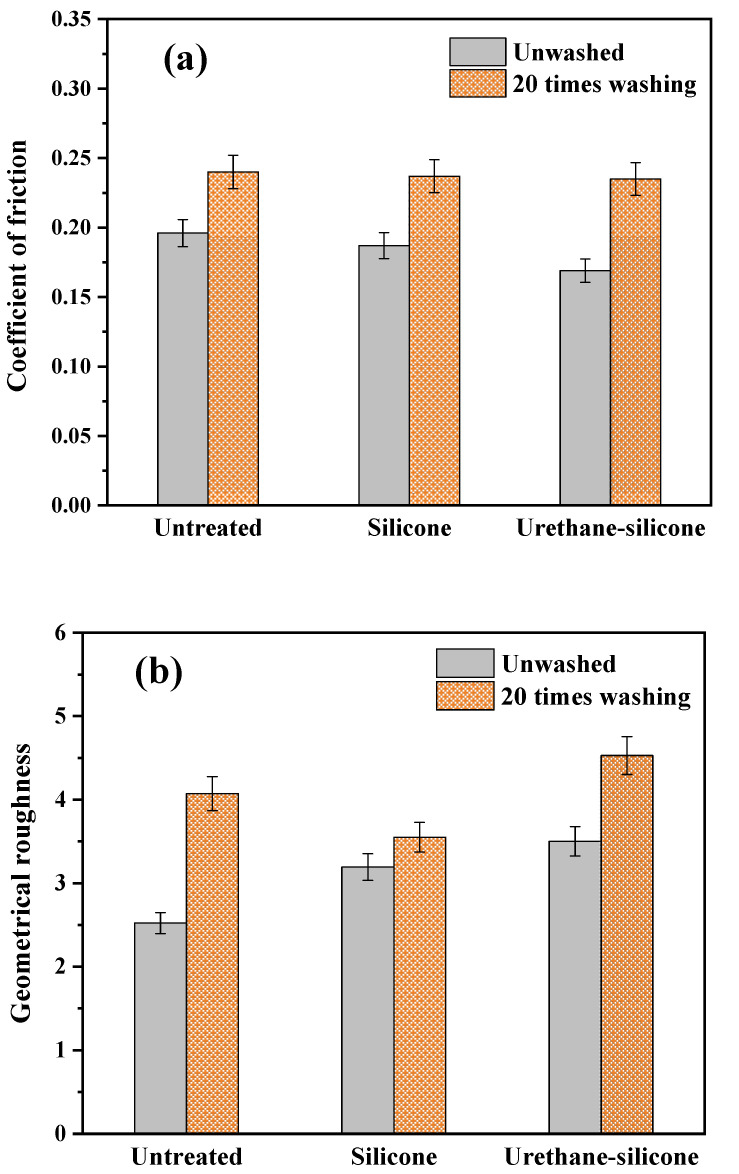
Surface properties of the cotton knit fabric untreated and treated with softeners. (**a**) Coefficient of friction; (**b**) geometrical roughness.

Figure 12 shows the coefficient of friction (MIU) measurements and geometrical roughness (SMD) measurements of the surface properties of the cotton knitwear treated with a softener. The first parameter, MIU, is correlated with the soft and smooth feeling when the surface of an object is touched; a smaller MIU value indicates a softer and smoother fabric. The SMD indicates the surface physical evenness; a higher value represents an uneven surface [49].

The MIU of the cotton knitwear treated with urethane–silicone softener was the smallest, showing the soft and smooth surface properties (Figure 12a). This is because the urethane–silicone softener has a small particle size on the nanoscale, and thus penetrates better into the inner structure of the fabric [15].

The SMD indicates surface irregularities, and urethane–silicone softener-treated cotton knitwear showed the highest value (Figure 12b). This is the result of a change in the geometry of the sample due to fabric shrinkage and the changes in thickness [50]. In addition, after washing 20 times, all three samples showed increased SMD values. It is thought that the chemical bond of the urethane–silicone softener increases the durability rather than the physical bond of a general softener, indicating strength in the smoothness of the surface [15].

## 4. Conclusions

This study examined the effect of using a reactive softener, and the washing durability of the cellulose-based cotton knitted fabric. For this purpose, a nanoscale softener was prepared by synthesizing a reactive urethane–silicone softener. The optimal reaction conditions for the urethane prepolymer for blocked isocyanate synthesis were determined to be 90 min at 90° C through content analysis of –NCO groups. Residual –NCO blocking of the urethane prepolymer was performed using low-temperature DMP at 70 °C for 60 min. The processability of the prepared urethane–silicone softener was evaluated using the KES-FB system after application to a cotton knit fabric. The chemical reaction between the surface of the cotton fabric and the silicone–urethane softener was effective in imparting dimensional stability in all physical properties. The cotton knitted fabric treated with the urethane–silicone softener exhibited soft and smooth surface properties due to the elasticity, flexibility, and restoring force against shear strength, as well as the restoring force against bending deformation and low coefficient of friction. These properties were maintained even after 20 washes. As a result, the prepared nanoscale reactive softener showed excellent performance in cotton knitwear. Therefore, it is expected to provide excellent performance and durability when applied to various cellulose fabrics.

## Figures and Tables

**Table 1:** Measurement parameter of the KES-FB system.

Properties	Symbols	Characteristic Values	Unit	Measuring Condition
Tensile properties	LT	Linearity	-	Max. Load: 50 gf/cm Tensile speed: 0.1 mm/s Measuring length: 2.5 cm
	WT	Tensile energy	gf·cm/cm^2^	
	RT	resilience	%	
Shear properties	G	shear stiffness	gf/cm·degree	Shear deformation: ±4°
	2HG	Hysteresis at ϕ = 4°	gf/cm	
Bending properties	B	Bending rigidity	gf·cm^2^/cm	Bending curvature (K): ±2.5 cm
	2HB	Hysteresis	gf·cm/cm	
Compression properties	T_0_	Thickness of fabric	mm	Max. Load: 10 gf/cm^2^ Compression speed: 50 mm/s Compression area: 2 cm^2^
	WC	Compressional energy	gf·cm/cm^2^	
Surface properties	MIU	Coefficient of friction	-	Friction static load: 50 g Roughness static load: 5 g
	SMD	Geometrical roughness	micron	

**Table 2:** Particle size of the blocked isocyanate emulsion.

Sample	Particle Size (um)
Blocked isocyanate emulsion	0.059213

## Data Availability

Data available in a publicly accessible repository.
